# Chronic stress of high dietary carbohydrate level causes inflammation and influences glucose transport through *SOCS3* in Japanese flounder *Paralichthys olivaceus*

**DOI:** 10.1038/s41598-018-25412-w

**Published:** 2018-05-09

**Authors:** Kangyu Deng, Mingzhu Pan, Jiahuan Liu, Mengxi Yang, Zhixiang Gu, Yue Zhang, Guangxia Liu, Dong Liu, Wenbing Zhang, Kangsen Mai

**Affiliations:** 1 The Key Laboratory of Aquaculture Nutrition and Feeds (Ministry of Agriculture), the Key Laboratory of Mariculture (Ministry of Education), Ocean University of China, Qingdao, 266003 China; 2 Laboratory for Marine Fisheries Science and Food Production Processes, Qingdao National Laboratory for Marine Science and Technology, Wen Hai Road, Qingdao, 266237 China

## Abstract

Carnivorous fish is thought to be high-glucose intolerance. But the reasons were still unclear. The aim of the present study is to investigate the effects of high level of dietary carbohydrate on the survival, growth and immune responses of *Paralichthys olivaceus*, and the underlying molecular mechanism related to the immune and glucose metabolism. *P. olivaceus* were fed with 8%, 16% and 24% of dietary carbohydrate for 10 weeks, respectively. After that, a glucose tolerance test (GTT) was conducted. Results showed that excessive (24%) dietary carbohydrate significantly decreased the growth and glucose tolerance ability according to the GTT. It significantly increased hepatic NADPH oxidase activity and malondialdehyde content and serum contents of IL-6 and advanced glycation end products. The expressions of glucose transport-relevant genes in liver and the content of related hormones in serum were analyzed. In conclusion, it was confirmed that IL-6 increased the expression of suppressor of cytokine signaling 3 (*SOCS3*) and regulated the downstream targets of PI3K-AKT mediated signal transduction, and then downregulated the glucose transporter 2 activity in liver of *P. olivaceus* fed diet with excessive carbohydrate level. It was suggested that *SOCS3* served as a bridge between immune response and glucose metabolism in *P. olivaceus*.

## Introduction

Carbohydrate is an excellent energy source for vertebrates, but cannot be fully and efficiently used by fish, especially for carnivorous fish species. Most carnivorous fish have been considered as glucose intorlerance and often display a prolonged postprandial hyperglycemia after oral or injected glucose loading and intake of carbohydrate-enriched diets^1–3^. In addition, fish showed high mortality, reduced growth performance, low nutrient utilization efficiencies and poor physiological functions, when administrated with excessive carbohydrate levels^4–8^. Many reasons were speculated to explain high-glucose intolerance in carnivorous fish, including a higher sensitivity of insulin to amino acids rather than glucose, inefficiencies in peripheral glucose utilization and absorption, inadequacies in homeostatic glucose regulation and imbalances of endogenous versus exogenous glucose sources^9–13^. However, there was no clearly conclusion at present.

Pronounced changes in glucose metabolism in fish could be induced by dietary carbohydrate levels. These changes included activities of the key enzymes involved in glucose metabolism, such as pyruvate kinase and glucokinase^14,15^. In previous study, genes of most of these enzymes have been cloned and characterized in fish. Meanwhile, the regulation of these enzymes at the molecular level by dietary carbohydrate manipulation has been reported^16–20^. However, most of the previous studies focused on the glycolysis and gluconeogenesis. Little research was reported on the molecular regulation of the upstream pathways.

Previous studies showed that the carnivorous fed high level of dietary carbohydrate could have suppressed immunity^21,22^. However, only some apparent parameters were analyzed, such as growth performance, mortality and activity of the immune related enzymes^23,24^. In mammals, it was confirmed that glucose metabolism was related to the immune response of animals, such as anti-oxidative capacity and inflammation^25–28^. Production of H_2_O_2_ has been shown to regulate insulin release to modulate proximal and distal insulin signaling^25,29^. At the same time, impaired immunity including ROS and inflammation could also affect inslulin signal pathway through activating numerous intracellular serine kinases and negative regulators of cytokine signaling, such as c-Jun N-terminal kinase (JNK) and suppressor of cytokine signaling (SOCS)^30–32^. Recent years, human diabetes was even regarded as a chronic inflammatory response^33^. So, it is worth to investigate if the high-glucose intolerance in fish is linked to the impaired immunity.

Japanese flounder (*Paralichthys olivaceus*) is a typical marine carnivorous fish species. After a 45-day feeing trial with different dietary carbohydrate sources, the previous study found that the Japanese flounder utilized dextrin more efficiently than glucose, and dextrin was a better source of energy than lipid. In that study, however, only the apparent parameters including growth performance, feed utilization and body compositions were analyzed^34^. The aim of the present study is to investigate the effects of high level of dietary carbohydrate on the survival, growth and immune responses of *P. olivaceus*, and the underlying molecular mechanism related to the immune and glucose metabolism.

## Results

### Survival and growth performance

**Figure 1:**
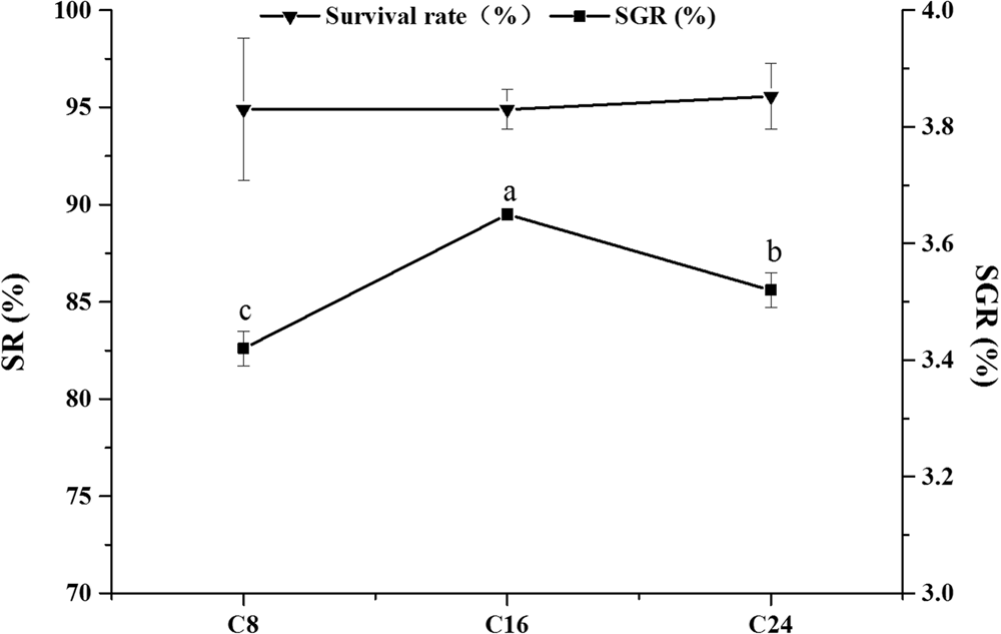
Survival rate (SR) and the specific growth rate (SGR) of Japanese flounder after the 10-week feeding trial. The values represent the means ± S.D., *n* = 3. Values with different letters mean significantly different (*P* < 0.05).

There were no significant differences in survival rate (SR, about 95%) among all the treatments (Fig. 1). With the increase of dietary carbohydrate levels, specific growth rate (SGR) significantly increased in the C16 group, and then decreased in the C24 group (p-value = 0.002). The lowest value of SGR was observed in the C8 group.

### Serum parameters after feeding trial

Data of the serum parameters are shown in Table 1. Compared with the C8 and C16 group, contents of total protein (TP), albumin (Alb), globulin (Glo), and advanced glycationend products (AGEs) in serum reached the significant highest values in the group of C24 (TP, p-value < 0.001; Alb, p-value = 0.024; Glo, p-value < 0.001; AGEs, p-value < 0.001). There were no significant differences in the content of tumor necrosis factor (TNF-α) and fasting serum insulin (FINS), activities of aspartate transaminase (AST) in serum among all the groups. The highest concentration of interleukin 6 (IL-6) in serum was found in C24 group, which was significantly higher than that in C16 group (p-value = 0.015). The activities of glutamic-pyruvic transaminase (ALT) and alkaline phosphatase (ALP) in serum significantly increased with dietary carbohydrate levels (ALT, p-value = 0.025; ALP, p-value = 0.045). There was no significant difference in the content of leptin between the group of C16 and C24. Both of them were significantly higher than that in C8 group (*P* < 0.001). There were no significant differences in concentration of adiponectin in serum among all the three groups.

**Table 1:** Serum parameters of Japanese flounder after the 10-week feeding trial.

Parameters	Diets		
	C8	C16	C24
Total protein (g/L)	25.13 ± 1.85b	26.73 ± 1.97b	37.40 ± 0.87a
Albumin (g/L)	4.25 ± 0.70b	4.13 ± 0.90b	6.20 ± 0.96a
Globulin (g/L)	20.88 ± 1.35b	20.75 ± 3.47b	31.20 ± 0.89a
IL-6 (pg/mL)	20.96 ± 2.63ab	19.56 ± 2.00b	24.51 ± 0.59a
TNF-α (pg/mL)	49.22 ± 7.79	51.33 ± 9.31	50.81 ± 1.71
ALT (U/L)	5.40 ± 0.36b	5.80 ± 0.42ab	6.40 ± 0.36a
AST (U/L)	12.90 ± 1.36	12.13 ± 1.69	12.55 ± 0.62
ALP (U/L)	61.65 ± 3.10b	64.68 ± 2.31ab	68.47 ± 3.41a
AGEs (mg/ml)	4.26 ± 0.11b	4.63 ± 0.23b	5.24 ± 0.23a
FINS (mIU/L)	21.33 ± 2.72	21.37 ± 2.04	21.89 ± 3.34
Leptin (ng/ml)	8.15 ± 0.50b	11.76 ± 0.74a	11.57 ± 0.80a
Adiponectin (µg/ml)	6.82 ± 1.29	7.09 ± 1.69	7.70 ± 1.39

Values are presented as mean ± S.D., *n* = 3 (8 fish/replicate). Values followed by different letters in the same row are significantly different (*P* < 0.05).

### Analysis of GTT

**Figure 2:**
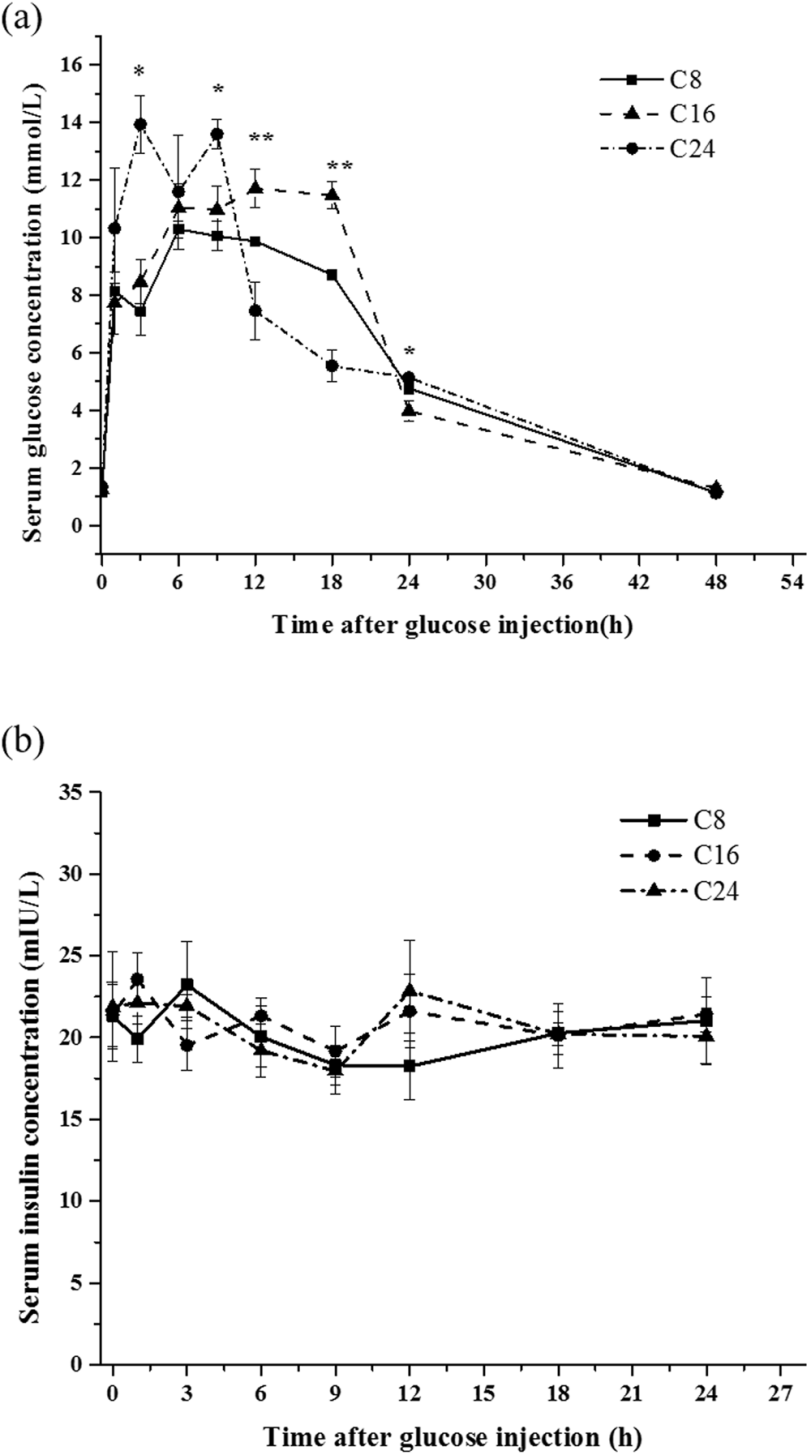
Response to glucose tolerance test of Japanese flounder after the 10-week feeding trial. (**a**) Serum glucose concentrations **(b**) Serum insulin concentrations. Values at 0 h are presented as mean ± S.D., *n* = 3 (8 fish/replicate) and the other values represented the means ± S.D., *n* = 3 (3 fish/replicate). * represents significant difference between C24 and C8 (*P* < 0.05); ** represents significant difference among all groups, (*P* < 0.05).

The fasting serum glucose (FSG) of the three groups were 1.15 ± 0.17, 1.25 ± 0.21 and 1.35 ± 0.12 mmol/L, respectively. In addition, there were no significant differences among all the three groups (Table 2). At the beginning, glucose injection induced a notable increase of the serum glucose level in all the groups, and then resulted in a treatment (C8, C16 and C24) × time (sampling time points after injection) interaction (Fig. 2a). During the test, serum glucose concentrations in all groups were significant higher than the FSG. At the 48th hour after injection, the serum glucose concentration in all the groups returned to the basal level.

**Table 2:** Analyzed parameters in the glucose tolerance test.

Parameters	Diets		
	C8	C16	C24
FSG (mmol/L)	1.15 ± 0.17	1.25 ± 0.21	1.35 ± 0.12
AUC_48_ ((mmol/L) × h)	219.27 ± 1.46	229.52 ± 4.19	222.82 ± 7.90
C_max_ glucose (mmol/L)	10.67 ± 0.35c	11.85 ± 0.32b	14.02 ± 0.13a
T_max_ (h)	7.35 ± 0.90a	7.65 ± 0.40a	3.21 ± 0.20b
CR_48_ (%/h)	1.29 ± 0.33b	2.38 ± 0.23a	0.27 ± 0.25c
T_basal_ (h)	37.22 ± 1.17b	33.32 ± 1.61b	45.72 ± 2.13a

Values of fasting serum glucose are presented as mean ± S.D., *n* = 3 (8 fish/replicate) and other values are presented as mean ± S.D., *n* = 3 (3 fish/replicate). Values followed by different letter in the same row are significantly different (*P* < 0.05).

The parameters of fitting equation (equation (3)) are shown in Table 3. Coefficients of all parameters were with 95% confidence bounds. The values of R square in all equations were over 0.84. According to the fitted equation, the max serum glucose concentration (C_max_) of serum peaked at 7.35 ± 0.90, 7.65 ± 0.40, 3.21 ± 0.20 hour with the C_max_ at 10.67 ± 0.35, 11.85 ± 0.32, 14.02 ± 0.13. No significant differences were found in areas under the curve of glucose during the 48 h of GTT (AUC_48_) among all the groups (Table 2). The highest value of clearance rate during the 48 h of GTT (CR_48_) was found in C16 group (2.38 ± 0.23%/h), and the lowest one was in C24 group (0.27 ± 0.25%/h). Compared with that in C24 group, the time return to basal serum glucose (T_basal_) was significantly shorter in C16 group (33.32 ± 1.61) (p-value = 0.000) and C8 group (37.22 ± 1.17) (p-value = 0.002).

**Table 3:** Parameters in the regression equations after the glucose tolerance test.

Diets	Parameters				
	a	b	c	d	R^2^
C8	18.02	−0.05038	−15.36	−0.2876	0.8523
	20.60	−0.05443	−17.97	−0.2546	0.8973
	32.01	−0.06802	−29.32	−0.1586	0.8489
C16	34.65	−0.07830	−31.80	−0.1876	0.8801
	39.42	−0.07570	−36.49	−0.1689	0.8966
	67.51	−0.09075	−65.05	−0.1434	0.8990
C24	18.10	−0.05837	−16.75	−0.9084	0.9287
	18.18	−0.05428	−16.85	−0.8265	0.9355
	17.43	−0.05542	−15.95	−0.9646	0.9309

Coefficients of all parameter are with 95% confidence bounds.

In the regard to the three groups, there were no significant differences in the serum insulin concentrations among all the sampling time points after injection of glucose. And there was no clear trend on the serum insulin concentrations changing with the sampling times (Fig. 2b).

#### Histological structure and oxidation resistance of liver

**Figure 3:**
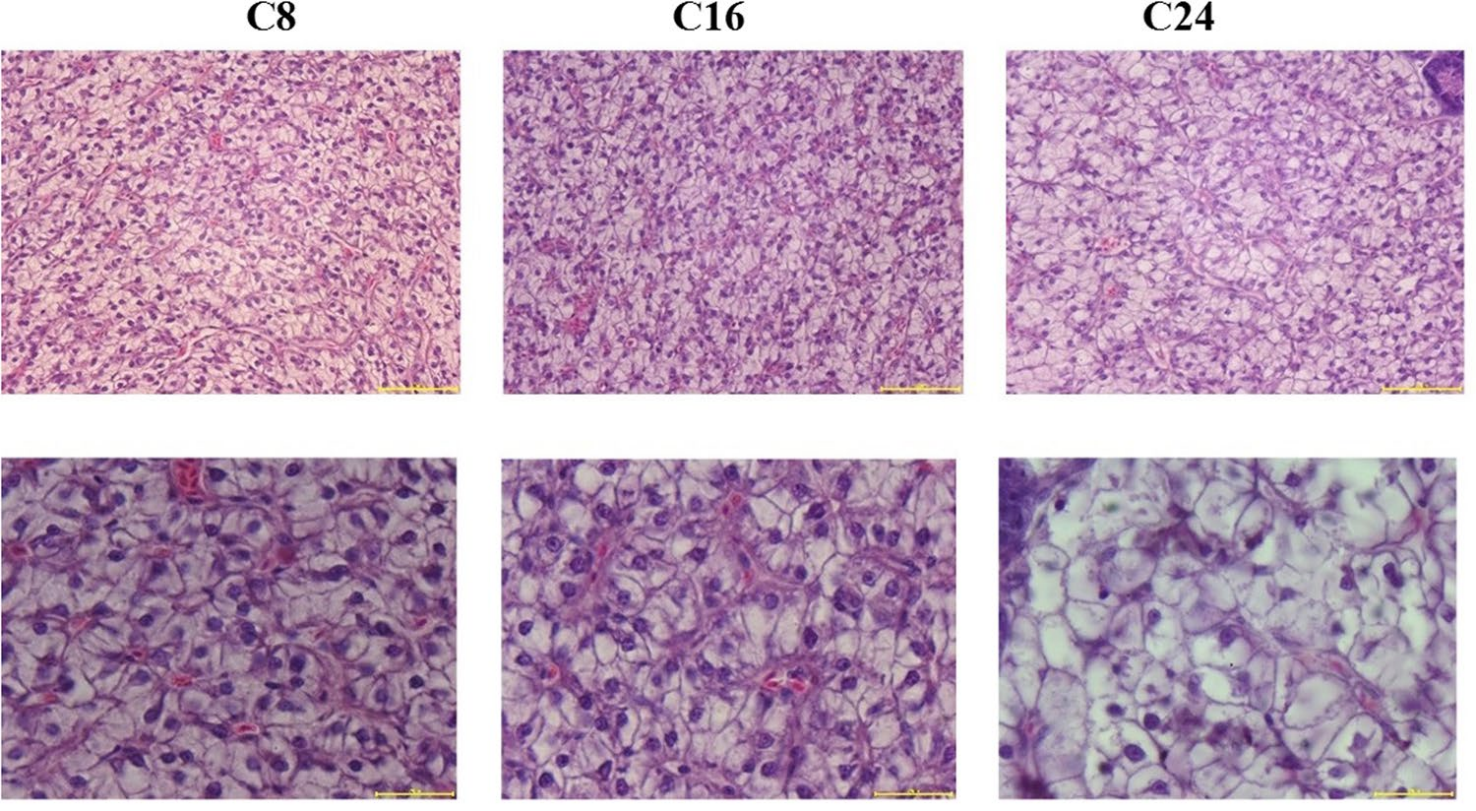
Effects of dietary carborhydrate levels on the hepatic histological characteristics of Japanese flounder after the 10-week feeding trial. Magnifications are ×400 (top) and ×1000 (bottom), and the scale bars are 20 μm (top) and 50 μm (bottom), respectively.

Hepatocytes from group C8 and C16 showed normal histology (Fig. 3). In C24 group, some hepatic cell outlines were indistinguishable and some nuclei were dissolved. Obvious hepatocytes swelling, nucleus polarization and lipid vacuolization were observed in C24 group.

The value of total anti-oxidative capacities (T-AOC) in liver in C24 group was significantly lower than those in C8 and C16 groups (Table 4) (p-value = 0.004). The C8 group had a significant higher superoxide dismutase (SOD) activity in liver than C24 group (p-value = 0.007). The C24 group had a significant higher malondialdehyde (MDA) in liver than C8 and C16 groups (p-value = 0.002). C16 group had a significant lower NADPH oxidase (NOX) activity in liver than C24 (p-value = 0.040), and there was no significant difference between C8 and C16.

**Table 4:** Anti-oxidative parameters in liver of Japanese flounder after the 10-week feeding trial.

Parameters	Diets		
	C8	C16	C24
T-AOC (U/mg protein)	0.66 ± 0.07a	0.82 ± 0.16a	0.41 ± 0.07b
SOD (U/mg protein)	1.45 ± 0.10a	1.30 ± 0.05ab	1.19 ± 0.04b
MDA (nmol/mg protein)	0.96 ± 0.09b	0.90 ± 0.07b	1.22 ± 0.08a
NOX (U/mg protein)	0.98 ± 0.05ab	0.90 ± 0.25b	1.65 ± 0.45a

Values are presented as mean ± S.D., *n* = 3 (8 fish/replicate). Values followed by different letters in the same row are significantly different (*P* < 0.05).

### Gene and protein expression in liver

**Figure 4:**
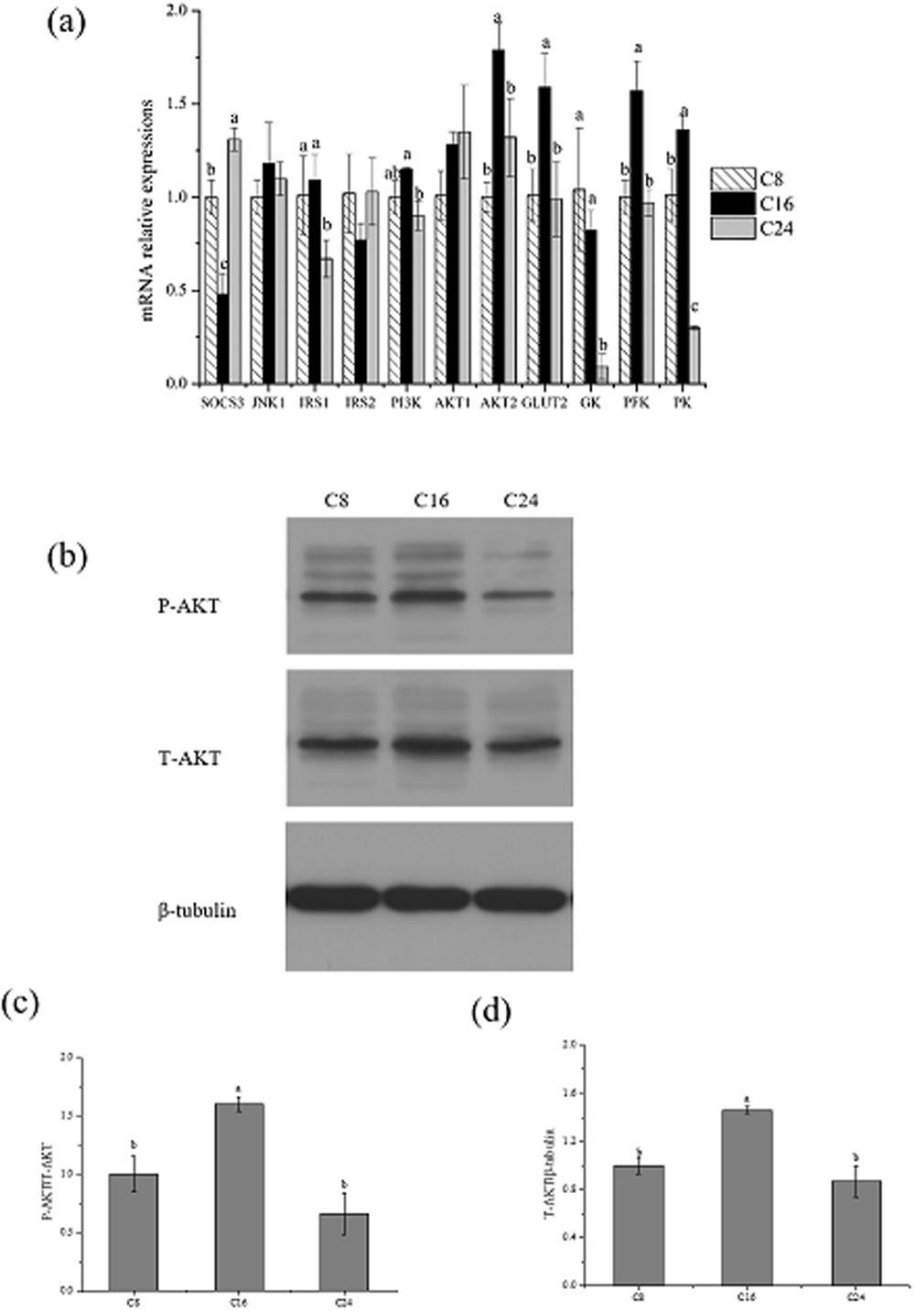
Gene and protein levels in liver of Japanese flounder after the 10-week feeding trial. (**a**) Expressions levels of glucose metabolism related genes were normalized to β-actin levels and expressed as relative expression values to those in C8 group. **(b)** The relative protein abundances of T-AKT and P-AKT in livers were expressed as relative expression values to those in C8 group. **(c,d)** The bars represent the mean ± S.D., *n* = 3 (8 fish/replicate). Values with different letters mean significantly different (*P* < 0.05). Unprocessed original scans of blots are shown in Supplementary Fig. 1.

The gene expressions of glucose utilization-relevant genes are shown in Fig. 4a. The mRNA levels of insulin receptor substrate 2 (*IRS2*), c-Jun N-terminal kinase 1 (*JNK1*) and protein kinase B 1 (*AKT1*) in liver had no significant differences among all the three groups. Gene expressions of the insulin receptor substrate 1 (*IRS1*) and glucokinase (*GK*) in C24 group was significantly lower than that in C16 group (*IRS1*, p-value = 0.039; *GK*, p-value = 0.003). Compared with C8, the mRNA level of *SOCS3* had the significant highest value in C24 group (p-value = 0.014), and the significant lowest value in C16 group (p-value = 0.001)). The mRNA level of phosphoinositide 3-kinase (*PI3K*), protein kinase B 2 (*AKT2*), glucose transporter 2 (*GLUT2*) and *phosphofructokinase* (*PFK)* had the significant highest value in C16 group (*PI3K*, p-value = 0.014; *AKT2*, p-value = 0.002; *GLUT2*, p-value = 0.008; *PFK*, p-value = 0.001), but no differences were found between C8 and C24. The mRNA levels of pyruvate kinase (*PK*) had the significantly higher and lower value in C16 and C24, respectively (p-value < 0.001). At the same time, phosphorylation of AKT (P-AKT) level in C16 was significantly higher than that in C24 (p-value = 0.009) and the total of AKT (T-AKT) in C16 was significantly higher than other two groups (T-AKT, p-value = 0.032) (Fig. 4b–d).

## Discussion

Based on the growth rate, the optimal levels of dietary carbohydrate for the carnivorous species, such as gilthead sea bream, giant croaker, Chinese long snout catfish, were determined less than 20%^15,35,36^. Instead, compared to that in the carnivorous species, the optimal levels for herbivorous species and omnivore species, such as gibel carp, white sea bream and grass carp, can be higher than 30%, even to 50%^37–39^. Excessive level of dietary carbohydrate could result in negative effects. In the present study, dietary carbohydrate supplementation at 24% led to reduced growth compared with 16% of dietary carbohydrate. Liver is a main metabolic organ. According to the histological analysis, the liver cell of Japanese flounder fed with 24% of dietary carbohydrate potentially indicated the impaired fuction of liver. In addtion, more oxidative damage in the liver of Japanese flounder was found in group with 24% of dietary carbohydrate compared with the other two groups. Based on these data, it was suggested 24% of dietary glucose concentration was overdose for Japanese flounder.

Impaired immunity in fish by overdose of dietary carbohydrate was observed in previous studies. It was reported that high level of dietary carbohydrate reduced plasma IgM and lysozyme levels in European whitefish^40^. The blood haemoglobin was negatively correlated with dietary carbohydrate levels in Atlantic salmon by long-term feeding of a high carbohydrate diet (20–30%)^24^. After the 10-week feeding trial, in the present study, the concentration of TP, Alb, Glo, AGEs and IL-6, the activities of ALP and ALT in serum and the content of MDA in liver significantly increased, while the activity of T-AOC and SOD in liver of Japanese flounder significantly decreased by the excessive dietary carbohydrate inclusion at 24%. This suggested that excessive dietary carbohydrate level could increase the oxidative stress and inflammation in Japanese flounder.

A fact was found that the dietary carbohydate intake affected the glucose tolerance in fish, while it seemed to have a poor ability to take care of excess glucose^41,42^. In mammals, fasting glucose concentration and fasting insulin concentration were used in calculating insulin sensitivity or resistance, in order to evaluate the glucose utilization ability. The relationship between blood glucose concentration in fish and dietary carbohydrate level was inconclusive. Hemre *et al*. found that blood glucose level in Atlantic salmon was correlated with dietary carbohydrate level^43^, while some studies reported serum glucose did not differ between the groups fed different dietary carbohydrate levels^24,44^. Insulin is a major pancreatic endocrine hormone, which regulates blood glucose levels and the underlying metabolic pathways in higher vertebrates. In the present study, FSG was not significantly affected by dietary levels. Meanwhile, the levels of circulating insulin kept the same level in Japanese flounder fed different levels of dietary carbohydrate in this study. This result was in accordance with an earlier study in rainbow trout, in which it was found that the insulin content in plasma of trout fed with the carbohydrate-enriched diet was not significantly increased when compared with that with carbohydrate-free diet^45^.

In fact, apart from FSG and in FINS, GTT is a more accurate way to evaluate the glucose utilization ability, where AUC of glucose was used to estimate the content of individual loaded glucose, CR and T_basal_ were used to judge the speed of glucose disappearance. A lower CR and longer T_basal_ with a large AUC were regared as impaired glucose tolerance^46–48^. GTT is required when humans have high fasting glucose levels but do not meet the diagnostic criteria for diabetes. GTT have been conducted in many fish species and is useful for evaluating glucose tolerance, such as rainbow trout^46^, channel catfish^49^ and white sturgeon^50^. In the present study, the mathematical modeling was used to evaluate the ability of glucose utilization during GTT in Japanese flounder after a 10-week feeding trial with different ditary carbohydrate levels. Consistent with the increased SGR, although no significant differences in AUCs were found, the fish fed with 16% of dietary carbohydrate had a significant higher CR and lower T_basal_. According to the data on growth and GTT, it was suggested that the ability of glucose utilization of Japanese flounder was improved when fed with 16% of dietary carbohydrate. And the feeding adapting was helpful for glucose tolerance.

Ability of glucose utilization is related to glucosesensing. Rainbow trout fed a diet with 20% of carbohydrate showed a higher postprandial glucose than that fed carbohydrate-free diet. This showed a positive response to the dietary carbohydrate based on the major components of glucosensing system including glucose transporters (GLUTs)^51^. Glucose metabolism depends on the intake of glucose. Insulin-induced PI3K-AKT signaling contributes to the regulation of GLUT2-mediated glucose uptake in liver. PI3K and AKT, which were two major nodes downstream of insulin receptor substrate (IRS), have been implicated in many of the metabolic actions of insulin, such as glucose transport. The rate-limiting enzymes in the glycolysis pathways, such as GK, PFK and PK, played key roles in glucose metabolism. Results in the present study showed that the upregulation of *PI3K*, *AKT2*, *GLUT2*, *PK* and *PFK* transcription and activies of the total AKT and AKT phosphorylation were found in C16 group. These could be helpful to explain why the Japanes flounder fed with 16% of dietary carbohydrate had better glucose utilization ability as shown by the data on growth and GTT.

Moreover, many studies in mammals have found that leptin and adiponectin could improve the glucose transport^52–54^, eventhough the detail was not clear. Fish definitely do have these hormones with some functional similarities and many other distinct features^55^. In the present study, there were no significant differences in concentration of adiponectin in serum among all the three groups. However, the concentrations of leptin in groups of C16 and C24 were significantly higher than that in C8 group. It was suggested that an increasing level of leptin not adiponectin improved the glucose transport in Japanese flounder. In the previous study in zebrafish, it was also found that leptin signaling regulates glucose homeostasis^56^. In the present study, however, Japanese flounder fed with higher dietary carbohydrate (24%) had a longer T_basal_ with lower CR, a dereasesd activety of PI3K-AKT and glycolysis pathways, a downregulated *GLUT2* transcription than those fed with 16% of dietary carbohydrate. This result implied that excessive dietary carbohydrate inhibited the ability of glucose transportation and glucose catabolism, which was not associated with the content of serum insulin, leptin and adiponectin.

Previous studies suggested many reasons to explain the high-glucose intolerance in fish, and many issues remained under debate. A growing number of studies indicated that the oxidative stress influences the insulin signal pathway in mammals^57,58^. Although these findings were reported in muscle cell, it is expected that they also occur in hepatocytes because the primary metabolic abnormalities in these cells also induce ROS production, thus affecting glucose utilization. AGEs accumulate under high-glucose conditions and affect ROS producing by NADPH oxidase^59–62^. In the present study, it was implied that ROS level increased in organism with a significant up-regulation in AGEs and NADPH oxidase in Japanese flounder fed diet with excessive carbohydrate level (24%). Houstis *et al*. pointed out that increases in ROS levels precede the onset of insulin resistance (IR) and might be causally linked to it^63^. It is still not clear exactly how oxidative stress causes insulin resistance or metabolic dysfunction in mammals. The possible mechanism suggested is that ROS could inhibit IRS to regulate the insulin signal pathway by c-Jun N-terminal kinase (JNK)^31,32^. JNK family members are encoded by three genetic loci, JNK1-3, JNK1 isoform is the major contributor to insulin resistance^64^. Activation of JNK1 has been shown to directly phosporylate IRS-1 at inhibitory sites that prevent recruitment of this protein to the activated insulin receptor^32^. In the present study, no significant difference in JNK1 gene expression was found although it was suggested that Japanese flounder fed with 24% of dietary carbohydrate was under oxidative stress. Another fact was found that inflammation can be induced by ROS through the induction of pro-inflammatory cytokines (e.g., TNF-α and IL-6), which are main key factors to affect glucose utilization via inducing liver insulin resistance^65–69^. TNF-α could also induce JNK to inhibit IRS^70^. In the present study, the serum TNF-α and hepatic *JNK1* gene expressions were not significantly affected by dietary carbohydrate levels, whereas IL-6 significanlty increased in C24 group compared with C16 group. These results indicated that TNF-α was not associated with the direct effect of carbohydrate. In fact, IL-6 could induce suppressor of cytokine signaling 3 (SOCS3) expression to affect insulin signaling^71^. SOCS3 is highly expressed in the liver and plays an important role in regulating IR^72^. The overexpression of SOCS3 can reduce insulin secretion by inhibiting preproinsulin gene transcription^73^. Furthermore, overexpression of SOCS3 decreased the hepatic expressions of IRS1, IRS2 and PI3K, and resulted in glucose intolerance and decrease glucose uptake^74,75^. In the present study, the observed differences in the gene expression levels of *SOCS3* and *IRS1* between Japanese flounder fed 24% and 16% carbohydrate. The downregulation gene expressions of downstream *PI3K*, *AKT2*, *GLUT2*, *GK, PFK* and *PK*, and total and phosphorylation of AKT level were found in C24 compared with C16. It was indicated that the high level of *SOCS3* level of Japanese flounder fed with 24% of dietary carbohydrate inhibited the glucose uptake through decreasing *IRS1* gene expression and the downstream PI3K-AKT signal way and thus affected glucose utilization. In addition, energy sensing was another factor could affect glucose utilization, and AMPK was an important energy sensor. In present study, the increasing digestable energy may result in the change of AMPK activity. The activation of AMPK was found to induce glucose uptake in trout myotubes^76^. In terms of nutritional regulation, high glucose intake was found to reduce the phosphorylation of AMPK in the liver of fish^77,78^. Moreover, the activity of AMPK was associated with imflammation. The upregulation of AMPK decreased the mRNA expression of proinflammatory^79^. Based on above researches, it can be speculate that the activity of AMPK decreasd and thus further promoted the inflammation. These data partly explained why the fish fed with 24% of dietary carbohydrate had lower ability of glucose utilization than those fed with 16% of dietary carbohydrate.

**Figure 5:**
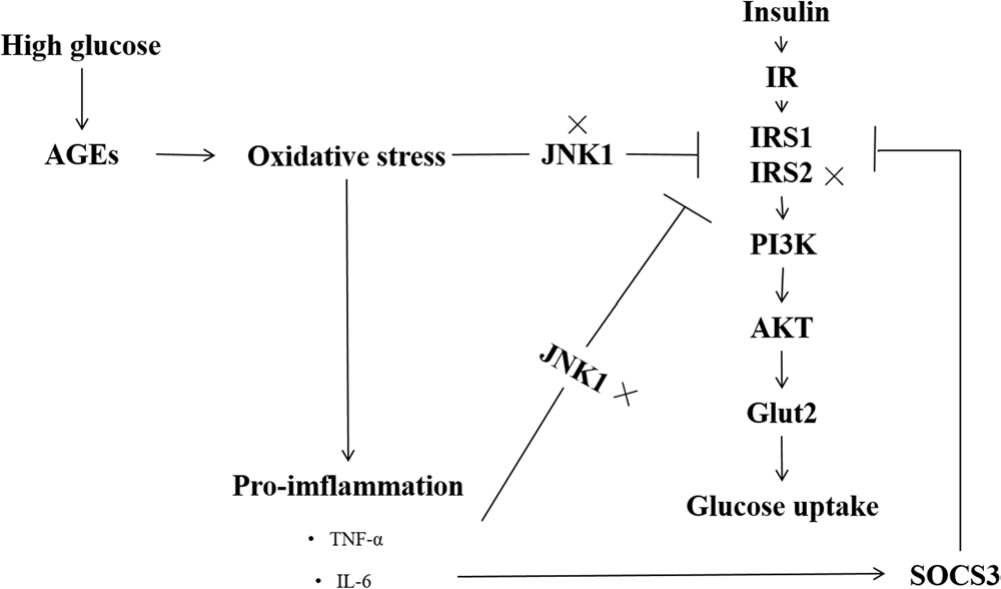
A potential reason of high gluose intolerance in Japanese flounder.

In conclusion, dietary carbohydrate is necessary for the growth and immune response of Japanese flounder. Diet with 16% of carbohydrate significantly improved the growth, glucose utilization and immunity of fish. And the feeding adapting was helpful for glucose tolerance. Excessive level (24%) of dietary carbohydrate caused oxidative stress, inflammation and influenced glucose transport. Based on the present data, it was suggested that SOCS3 served as a bridge between immune response and glucose metabolism. IL-6 increased the gene expression of *SOCS3* and thus regulated the downstream targets of PI3K-AKT mediated signal transduction that underlie the downregulation of *GLUT2* activity, glucose uptake and metabolism in liver. This hypothesis was illustrated in Fig. 5.

## Materials and Methods

### Experimental diet

Diet formulation and its proximate composition are given in Table 5. Three isonitrogenous and isolipidic diets are formulated with graded levels of carbohydrate (8%, 16% and 24%, respectively). They were named as C8, C16 and C24, respectively. All ingredients were finely ground, well mixed, and dry extruded in a laboratory pellet mill (EL220, Shangdong Haiyang, China). The diameters of the diet particles were 3 mm and 5 mm die. The particles were dried in a forced air oven at 50 °C for 8 h and stored in a refrigerator (−20 °C) until used.

**Table 5:** Formulation and proximate chemical composition of diets.

Ingredients (%)	Diets		
	C8	C16	C24
Fish meal	57	57	57
Wheat gluten	13	13	13
Alpha-starch	5	5	5
Corn starch	1.7	9.7	17.7
Soybean lecithin	1	1	1
Fish oil	3.5	3.5	3.5
Choline chloride	0.4	0.4	0.4
Ethoxyquin	0.05	0.05	0.05
Mold inhibitor	0.1	0.1	0.1
Monocalcium phosphate	0.5	0.5	0.5
Vitamin premix^a^	0.6	0.6	0.6
Minerals premix^b^	0.5	0.5	0.5
Carboxymethyl cellulose	16.65	8.65	0.65
***Proximate analysis***			
Dry matter (DM), % diet	96.80	97.23	96.96
Crude protein, % DM	49.59	49.60	50.04
Crude lipid, % DM	9.73	9.98	9.96
Reducing sugar, % DM	7.01	15.60	23.18
Ash, % DM	12.46	11.73	12.13
Gross energy (GE), kJ/g	18.93	19.06	19.20

^a^Vitamin premix (g kg^−1^ of mixture): microcrystalline cellulose, 16.473; VA, 0.032; VB_1_, 0.025; VB_2_, 0.045; VB_6_, 0.02; VB_12_, 0.01; VD, 0.035; VE, 0.24; VK, 0.01; calcium pantothenate, 0.06; nicotinic acid, 0.2; folic acid, 0.02; biotin, 0.06; inositol, 0.8; VC phosphate, 2.

^b^Mineral premix (g kg^−1^ of mixture): MgSO_4_·7H_2_O, 1.2; CuSO_4_·5H_2_O, 0.01; FeSO_4_·H_2_O, 0.08; ZnSO_4_·H_2_O, 0.05; MnSO_4_·H_2_O, 1.2; CuSO_4_·5H_2_O, 0.01; FeSO_4_·H_2_O, 0.08; ZnSO_4_·H_2_O, 0.045; CoCl_2_·6H_2_O (1%), 0.050 Na_2_SeO_3_ (1%), 0.02; calcium iodate, 0.06; zeolite powder, 8.485.

### Animals and feeding trial

Japanese flounders were obtained from Haiyang, Shandong Province, China. They (initial body weight: 7.14 ± 0.10 g) were randomly distributed into 9 tanks (3, 000 L) in a flow-through water system supplied with sand filtered seawater at a flow rate of 2.5 L/min. Three tanks were used for one treatment (150 fish/tank). The fish were fed to apparent satiation twice daily (8:00 and 18:00) for 10 weeks. During the feeding trial, the dissolved oxygen content was approximately 7.4 mg/L. Water temperature naturally ranged from 21 to 24 °C. The photoperiod was maintained at intervals of 13-h light: 11-h dark. All procedures performed in study were in strict accordance with the recommendations in the Guide for the Use of Experimental Animals of Ocean University of China. The protocols for animal care and handing used in this study were approved by the Institutional Animal Care and Use Committee of Ocean University of China.

### Sampling

At the end of the 10 weeks feeding trial, all the fish were fasted for 24 h after the last feeding and anaesthetized with MS-222 (Sigma, 50 mg L^−1^ water) before sampling. Fish in each tank were weighed and counted to determine SGR and SR. Eigth fish per tank were sampled to collect blood from coccygeal vertebra vein. The serum was separated and stored at −80 °C until analysis^80,81^. After that, these fish were dissected to collect liver. And the livers were quick frozen using liquid nitrogen and stored at −80 °C for subsequently analysis.

### Glucose tolerance test

At the end of the feeding trial, the serum of fish starved for 24 h was used to measure FSG and FINS concentration, which were setted as a baseline for AUC of glucose calculation and for comparison with insulin content after GTT, respectively. Another 30 anaesthetized fish per tank were used for GTT according to the method of López-Olmeda^82^. Anaesthetized fish per tank were intraperitoneal injected with glucose at a concentration of 600 mg per kg fish. After that, the blood was taken at 1, 3, 6, 9, 12, 18, 24 and 48 h after injection, respectively, to analyze serum glucose and insulin concentrations. Three fish per tank were used for collecting the serum at each sampling time point. The AUC of glucose, CR, C_max_, T_basal_ and the time reach the max serum glucose concentration (T_max_) were calculated.

### Serum and liver parameters

The concentrations of glucose, TP, Alb and Glo, and the activities of ALP, ALT and AST in serum were analyzed by the automatic biochemical analyzer (AU5400, Beckman). The concentration of insulin, leptin, adiponectin, IL-6, AGEs and TNF-α in serum were determined using a double antibody sandwich enzyme-linked immunosorbent assay. Kits for insulin, leptin, IL-6 and TNF-α were purchased from Cusabio, China. Kits for adiponectin and AGEs were purchased from Mybiosource, USA. All these kits were developed using antigenic regions completely conserved in fish^83–86^. The protein concentration, T-AOC, MDA, and SOD activity in liver were measured by commercial kits (Nanjing Jiancheng Bioengineering Institute, China)^87–89^. And the activity of NOX in liver was detected according to Li *et al*.^89^.

### Histological analysis

Liver samples were fixed in 10% neutral buffered formalin for 24 h and then transferred to 70% ethanol. Samples were then dehydrated through graded levels of ethanol (70%, 80%, 85%, 90%, 95%, 100%) using a tissue processor, cleared and embedded in paraffin wax (PPDT-12C1, Ceike, China). Transverse sections (~7 μm) were prepared using a rotary microtome (RM2235, Leica, China), mounted onto slides and processed for staining (Thermo Gemini A2, USA) with hematoxylin and eosin (H&E, Nanjing Jiancheng Bioengineering Institute, China). Slides were viewed under light microscopy and photographed with a digital camera (Olympus, DP72, Nikon, Japan).

### Real time PCR

Total RNA was extracted from 0.1 g of liver tissue using Trizol Reagent (Invitrogen, USA). It was then quantified and the purity was assessed by spectrophotometry. The 260:280 ratios were 1.8 ~ 2.0. Complementary DNA (cDNA) was synthesized from 1 mg of total RNA using PrimeScript^®^ RT reagent Kit with gDNA Eraser (Takara, Japan). Expressions of the selected genes were analyzed by the real-time PCR, which was performed with an ABI 7500 instrument (Applied Biosystems) using SYBR Green PCR (Takara). The Reaction mixtures (SYBR^®^ Premix Ex Taq^TM^ II (2×) 10.0 μl, Forward Primer (10 μM) 0.8 μl, Reverse Primer (10 μM) 0.8 μl, DNA template 2.0 μl and ddH_2_O 6.4 μl) were programmed 30 s at 95 °C, followed by 40 cycles of 5 s at 95 °C, 30 s at 58 °C, 30 s at 72 °C, and finally 15 s at 95 °C, 1 min at 60 °C and 15 s at 95 °C. For each mRNA, gene expression was corrected by *β-ACTIN* in each sample. The data were analyzed by the ΔΔCt method, and the primers used are shown in Table 6.

**Table 6:** List of PCR primer pairs used for the real-time PCR analysis.

Primers	Forward (5′-3′)	Reverse (5′-3′)
*SOCS3*	TTTCTTCACCCTGTCCGTGC	CCAGCCCTTTCCCCATGTAG
*JNK1*	TGGTCCGGGGTAGTGTGTTG	TCTCTGGCTTGGCTCGCTTT
*PI3K*	GCTCATCAACCACTATCGC	TGTCTTCTTTCACCACCTG
*IRS1*	CCCACTTAGGAAAAGCAGAG	AGTACAGGAACGGAAGGATC
*IRS2*	GGAGGTATGGCAAGTGAAT	AAGAAGAAGCTGTCGGAGT
*AKT1*	GAGGGAAGAATGGACGAAAG	TTCCCAGGAGTTTGAGGTAT
*AKT2*	CATCCCTTTCTAACAACACTA	CTGTAAACAACATTGCGTGA
*GLUT2*	GAACAGCACAGAAGAAGAGG	ACAGCCAGAACATTGACCAT
*PK*	GCTSGACTACAAGAACATC	CTCGTGGTTCTCCAGYTTG
*GK*	GGGATGATTGTTGGCACT	TGGAACCTGTCACGGAAA
*PFK*	TTGTAATCGGAGGGTTCG	ATTGTTGCTGATGGTGGC
*β-ACTIN*	GGAAATCGTGCGTGACATTAAG	CCTCTGGACAACGGAACCTCT

### Western blot analysis

Tissues were homogenized with glass Tenbroeck tissue grinders (Kimble Chase, USA) on ice and lysed with 50 mM Tris·HCl, 150 mM NaCl, 0.5% NP-40, 0.1% SDS, and 1 mM EDTA, pH 7.5, with protease and phosphatase inhibitor cocktails (Roche, Switzerland) at 4 °C for 1 h and cleared by centrifugation at 12,000 g for 20 min. Protein concentrations were determined with a BCA protein assay kit (Beyotime Biotechnology, China) using bovine serum albumin as standard. Protein samples (30 μg protein per lane) were separated by SDS-PAGE and transferred to 0.45 μm PVDF membrane (Millipore, USA) for Western analysis. The membrane was blocked with 5% nonfat milk in TBST buffer (20 mM Tris·HCl, 500 mM NaCl, 0.1% Tween 20) for 1 h at room temperature and incubated with primary antibody overnight at 4 °C before horseradish peroxidase (HRP)-conjugated secondary antibodies were added and visualized using ECL reagents (GoodHere, China). The following antibodies were used: antibodies against protein kinase B (AKT, 9272), phospho-AKT (Ser473, 9271) and β-tubulin (2146) were purchased from Cell Signaling Technology. All these antibodies were developed using antigenic regions completely conserved in fish, and could be used in scophthalmidae turbot^90^. The Western bands were quantified using NIH Image 1.63 software.

### Calculation and statistical analysis

The following parameters were calculated as:

1 $${\rm{Survival}}\,{\rm{rate}}\,({\rm{SR}}, \% )=100\times {\rm{final}}\,{\rm{fish}}\,{\rm{number}}/{\rm{initial}}\,{\rm{fish}}\,{\rm{number}}$$

2 $${\rm{Specific}}\,{\rm{growth}}\,{\rm{rate}}\,({\rm{SGR}}, \% )=100\times [(\mathrm{ln}\,{\rm{final}}\,{\rm{body}}\,{\rm{weight}}-\,\mathrm{ln}\,{\rm{initial}}\,{\rm{body}}\,{\rm{weight}})\times {{\rm{days}}}^{-1}$$

The PROC NLIN was used to fit exponential curves for glucose concentration during 48 hours GTT, using the following equation performed by Metlab2017:

3 $${\rm{F}}({\rm{t}})={\rm{a}}\times \exp \,(\,-\,{\rm{b}}\times {\rm{t}})+{\rm{c}}\times \exp \,(\,-\,{\rm{d}}\times {\rm{t}});$$

where F (t) was the metabolite concentration at time t (hour); the method was modified based on Pires *et al*.^91^. CR (%/h), C_max_ (mmol/L), T_basal_ (h) and T_max_ (h) were calculated as the actual equation. CR was calculated as: clearance rate

4 $$({\rm{CR}};\, \% /h)=\{(\mathrm{ln}\,[{\rm{ta}}]-\,\mathrm{ln}\,[{\rm{tb}}])/({\rm{tb}}-{\rm{ta}})\}\times 100;$$

where [ta] was the concentration of glucose at time a (ta), and [tb] was the concentration of glucose at time b (tb). The AUC was calculated using the trapezoidal method and actual concentration values, after discounting the baseline concentration (FSG, calculated by averaging value before injection)^87^.

All statistical evaluations were analyzed using the software SPSS 19.0. The growth parameters, immune parameters and gene expression were analyzed using one-way analysis of variance (ANOVA) followed by Tukey’s multiple range tests. The effects of time and diets and their interactions were analyzed by two-way ANOVA. Tukey’s multiple range tests were used to examine treatment differences among the interactions. When the interaction was significant, the results were further analyzed using one-way ANOVA and Tukey’s multiple range test. In case unequal variance was determined by Levene’s test, the data were square root-transformed before statistical analysis. Differences were regarded as significant when *P* < 0.05. All data were expressed as means ± standard deviations.

### Data availability

The datasets generated and analysed during the current study are vailable from the corresponding author on reasonable request.

## Electronic supplementary material

Supplementary file
