# Supplementary material for: Chronic stress of high dietary carbohydrate level causes inflammation and influences glucose transport through SOCS3 in Japanese flounder Paralichthys olivaceus
Source: Sci Rep. 2018 May 9;8:7415. doi: 10.1038/s41598-018-25412-w (PMC5943576; doi:10.1038/s41598-018-25412-w)

Chronic stress of high dietary carbohydrate level causes inflammation and influences glucose transport through *SOCS3* in Japanese flounder *Paralichthys olivaceus*

Kangyu Deng[1], Mingzhu Pan[1], Jiahuan Liu[1], Mengxi Yang[1], Zhixiang Gu[1], Yue Zhang[1], Guangxia Liu[1], Dong Liu[1], Wenbing Zhang[1, 2 *], Kangsen Mai[1, 2]

[1] The Key Laboratory of Aquaculture Nutrition and Feeds, Ministry of Agriculture, The Key Laboratory of Mariculture, Ministry of Education, Ocean University of China, Qingdao 266003, China.

[2] Laboratory for Marine Fisheries Science and Food Production Processes, Qingdao National Laboratory for Marine Science and Technology, Wen Hai Road, Qingdao 266237, China.

Supplementary data file

Contents:

Legends to supplementary figure 1

Supplementary figure 1

Legends

Supplementary Figure 1. Scans of developed films with approximate regions used for figures marked with rectangles and molecular sizes indicated in kDa (based on prestained protein markers).

Supplementary figure 1

Figure 4b

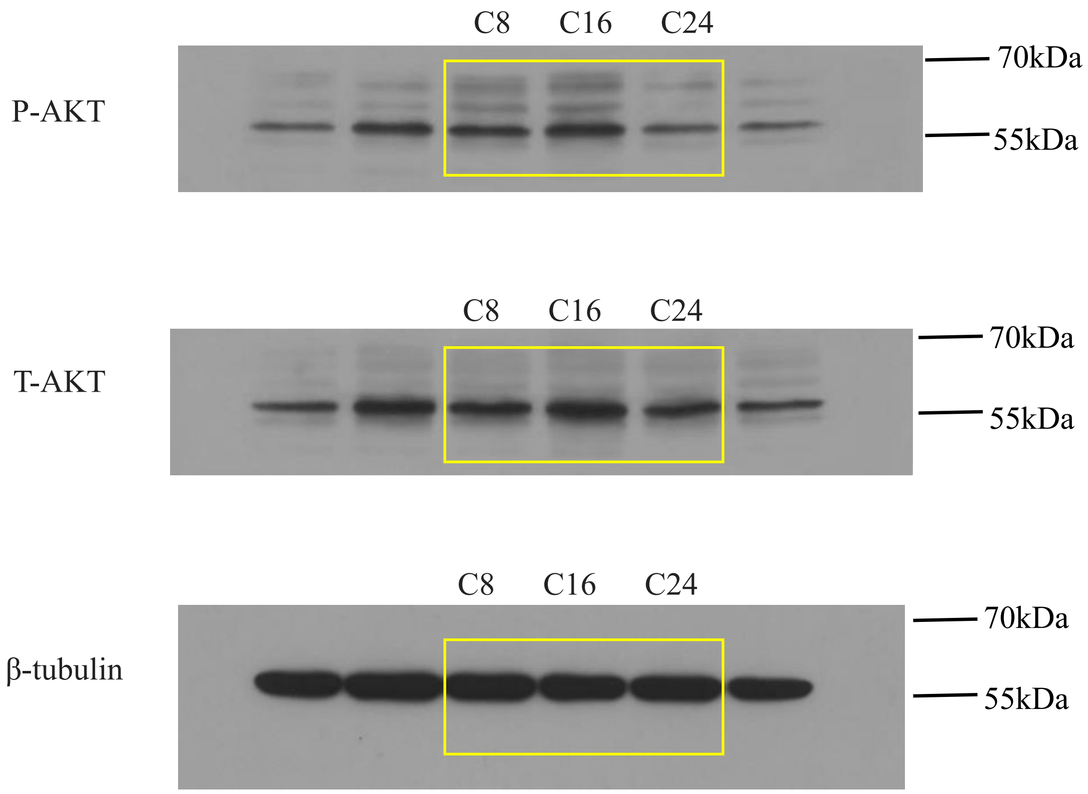

Supplement: Supplementary file 1 — Supplementary file [file 41598_2018_25412_MOESM1_ESM.pdf]
